# Postfunctionalization of Alkyne-Linked Conjugated Carbazole Polymer by Thermal Addition Reaction of Tetracyanoethylene

**DOI:** 10.3390/ma3104773

**Published:** 2010-10-15

**Authors:** Tsuyoshi Michinobu, Hiroyuki Fujita

**Affiliations:** 1 Global Edge Institute, Tokyo Institute of Technology, 2-12-1 Ookayama, Meguro-ku, Tokyo 152-8550, Japan; 2 PRESTO, Japan Science and Technology Agency (JST), 4-1-8 Honcho, Kawaguchi, Saitama, Japan; 3 Department of Organic and Polymeric Materials, Tokyo Institute of Technology, 2-12-1 Ookayama, Meguro-ku, Tokyo 152-8552, Japan

**Keywords:** addition reaction, charge-transfer band, conjugated polymer, electrochemistry, postfunctionalization

## Abstract

The postfunctionalization of the main chain alkyne moieties of carbazole containing poly(arylenebutadiynylene)s was attempted by using a high yielding addition reaction between electron rich alkynes and a strong acceptor molecule, tetracyanoethylene (TCNE). After successful postfunctionalization, the polymer band gap decreased due to the intramolecular donor-acceptor interactions. The resulting donor-acceptor alternating polymer showed a very broad charge-transfer band in the visible region as well as redox activities in both anodic and cathodic directions. The optical band gap showed good agreement with the electrochemical band gap. Furthermore, the thermal stability was enhanced after postfunctionalization. These features of the donor-acceptor alternating polymer are expected to be useful for high performance activities in organic solar cell applications.

## 1. Introduction

Since the discovery of conducting polyacetylene films, organic conjugated polymers have become an important class of materials for applications in the next generation of organic electronic devices [1]. Introduction of solubilizing substituents, such as alkyl chains, is a general method for improving polymer solubility, which enables preparation of thin films by low cost wet processes.

Recent trends of conjugated polymer development have been directed toward the design of more complex monomer repeat units. For example, donor-acceptor type conjugated polymers are composed of donor-acceptor repeat units and are now recognized as promising p-type semiconducting polymers for applications in bulk heterojunction organic solar cells [2]. These polymers are usually mixed with n-type semiconducting materials, such as fullerene derivatives, and the mixtures are spin-coated or screen-printed onto transparent conducting electrodes, such as ITO (indium tin oxide). Then, a lower work function metal, such as aluminum, is vacuum deposited as a top electrode. There are indeed many factors affecting the device performance, but it is apparent that both p-type and n-type semiconducting materials forming the active layers are the key to improve performance.

A conventional synthetic method for achieving donor-acceptor type conjugated polymers is based on the metal catalyzed polycondensation between a bifunctional electron donating monomer and a bifunctional electron accepting comonomer [3]. This technique is able to strictly control the sequential alternation of donor and acceptor units within a single polymer chain. However, there are, to our knowledge, no other techniques to prepare donor-acceptor alternating conjugated polymers.

**Scheme 1:**
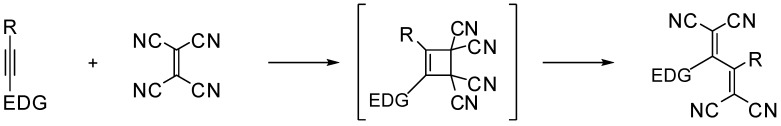
A high yielding addition reaction between alkynes activated by an electron-donating group (EDG) and tetracyanoethylene (TCNE).

We recently started a new project on the synthesis of donor-acceptor type polymers by a novel postfunctionalization method based on a high yielding addition reaction between electron rich alkynes and strong acceptor molecules, such as tetracyanoethylene (TCNE) [4]. The reaction mechanism is shown in Scheme 1. The thermal [2+2] cycloaddition between alkynes and electron deficient ethene moiety of TCNE is followed by the ring opening to provide the donor-acceptor chromophores [5]. The alkyne reactivity depends on the electron-donating substituents. As stronger donor groups are employed, higher yields are generally achieved. Thus, aromatic amines are one of the most suitable donor groups for achieving high yields in this addition reaction [6,7,8,9,10]. We have already demonstrated the quantitative postfunctionalization of aromatic polyamines substituted by electron rich alkynes as a side chain [11,12,13,14]. As more TCNE were added, the thermal stability and n-type character of the aromatic polyamines was significantly enhanced.

In this article, we apply the high yielding TCNE addition reaction to aromatic polyamines containing main chain alkynes and show for the first time the postfunctional synthesis of purely organic donor-acceptor alternating conjugated polymers [15].

## 2. Results and Discussion

### 2.1. Polymer Synthesis

The precursor polymers, poly[(*N*-hexadecyl-3,6-carbazolylene)butadiynylene] **P1** and poly[(*N*-hexadecyl-2,7-carbazolylene)butadiynylene] **P3**, were prepared by the acetylenic oxidative coupling of diethynyl-*N*-hexadecylcarbazole derivatives under the Hay conditions (O_2_, CuCl, *N*,*N*,*N*’,*N*’-tetramethylethylenediamine, toluene) [16,17,18]. The hexadecyl substituent was employed as a solubilizing group. Thus, **P1** and **P3** showed good solubilities in common organic solvents and the molecular weight was determined by GPC using THF as an eluent. The weight-average molecular weight (*M*_w_) and polydispersity (*M*_w_/*M*_n_) of **P1** were 10500 and 1.4, respectively, and those of **P3** were 18000 and 2.0, respectively.

**Scheme 2:**
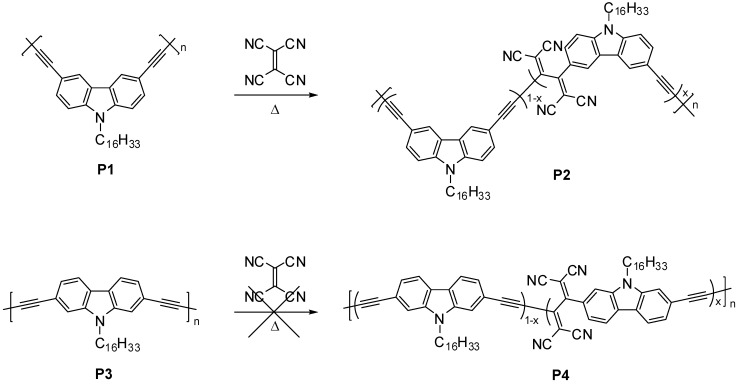
Postfunctionalization of alkyne-linked conjugated carbazole polymers by TCNE addition reaction.

The postfunctionalization reaction of **P1** and **P3** was performed in 1,2-dichlorobenzenze at 100 °C (Scheme 2). The reaction temperature was determined so as not to incur any decomposition of both precursor and TCNE adducted polymers on the basis of the thermogravimetric analysis (*vide infra*). During the reaction, the solution color of **P1** changed from yellow to red, suggesting progression of TCNE addition. In contrast, no TCNE addition occurred at the alkynes of **P3**. This difference can be explained by the substitution pattern of the carbazole moiety. The alkynes of **P1** are located at the *p*-phenylene positions relative to the nitrogen atom, while those of **P3** are at the *m*-phenylene positions. The former alkynes were activated by the electron-donating nitrogen atom, while the activation of the latter alkynes was not sufficient due to the cross-conjugation [19,20,21]. This result is consistent with previous systematic studies on small alkyne molecules [7] and conjugated carbazole polymers [22,23,24,25,26,27,28].

**Figure 1:**
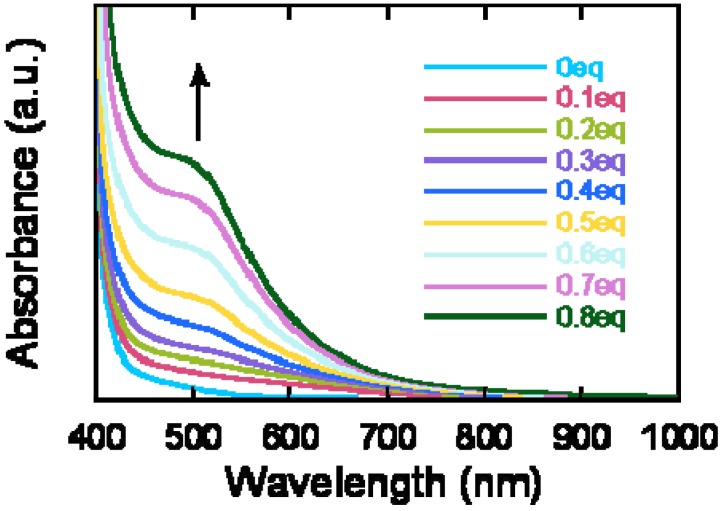
UV-Vis titration experiments of **P1** in 1,2-dichlorobenzene with a TCNE solution in 1,2-dichloroethane at 100 °C. Reaction completion was confirmed by time-dependent measurements.

UV-vis spectra provide a good guide to estimate the degree of the TCNE addition. A titration experiment of **P1** with TCNE was performed under the same reaction conditions (in 1,2-dichlorobenzenze, 100 °C) and the UV-vis spectral change was monitored as a function of the TCNE addition amount (Figure 1). A solution of the precursor polymer **P1** showed a yellow color with the longest absorption wavelength *λ*_max_ of 385 nm. After the TCNE addition, a new charge-transfer (CT) band appeared at *circa* 515 nm, leading to a red solution color. This CT band intensity increased with the increasing amount of TCNE addition, and the increase was saturated at the TCNE addition amount of 0.8 equiv. relative to the repeat unit. This result suggested that the donor-acceptor alternating conjugated polymer **P2**, with up to 0.8 of the donor-acceptor chromophore content *x*, can be prepared under the employed reaction conditions.

### 2.2. Characterization

The donor-acceptor alternating polymer **P2** was prepared by heating the **P1** solution to 100 °C for 48 h in the presence of 1.0 equiv. TCNE followed by the removal of unreacted TCNE *in vacuo*. It should be noted that no tedious purification processes are necessary. Furthermore, since there are no byproducts, the TCNE addition amount can be calculated from the weight increase (gravimetric analysis). The calculated TCNE addition yield was 71.6%. Elemental analysis of **P2** also gave the donor-acceptor chromophore content (*ca*. 60%), which was slightly lower than that determined by the gravimetric analysis. However, both values are in fair agreement within an experimental error. All these results suggested that TCNE addition reaction proceeded smoothly under mild conditions in relatively high yields.

**Figure 2:**
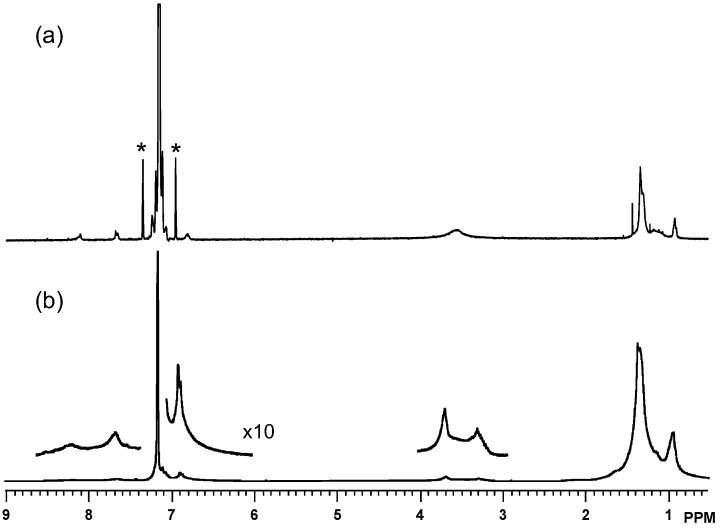
^1^H NMR spectra of (**A**) **P1** and (**B**) **P2** in CDCl_3_ at 20 °C. The spinning side bands of the solvent peak are marked.

The ^1^H NMR spectrum of **P1** displayed the well-defined peaks consistent with the chemical structure. In addition to the alkyl chain attached to the carbazole nitrogen atom appearing at 0.93–1.44 and 3.56 ppm, three kinds of aromatic protons were detected at 6.81, 7.67, and 8.11 ppm (Figure 2a). As compared to the precursor polymer, the spectrum of **P2** was slightly broadened (Figure 2b). Enlargement of the aromatic protons revealed a more complex structure. Furthermore, the methylene protons attached to the carbazole nitrogen atom were split due to the two different chemical environments, namely the TCNE adducted moiety and unreacted moiety. The higher field peak at 3.29 ppm was supposed to have derived from the TCNE adducted repeat unit, while the lower field peak at 3.68 ppm originated from the unreacted repeat unit. However, the content of each repeat unit could not be determined from the peak integration, due to the different broadening extent and the partial overlap. The ^13^C NMR spectrum of **P2** also did not provide useful information due to the broadening.

**Figure 3:**
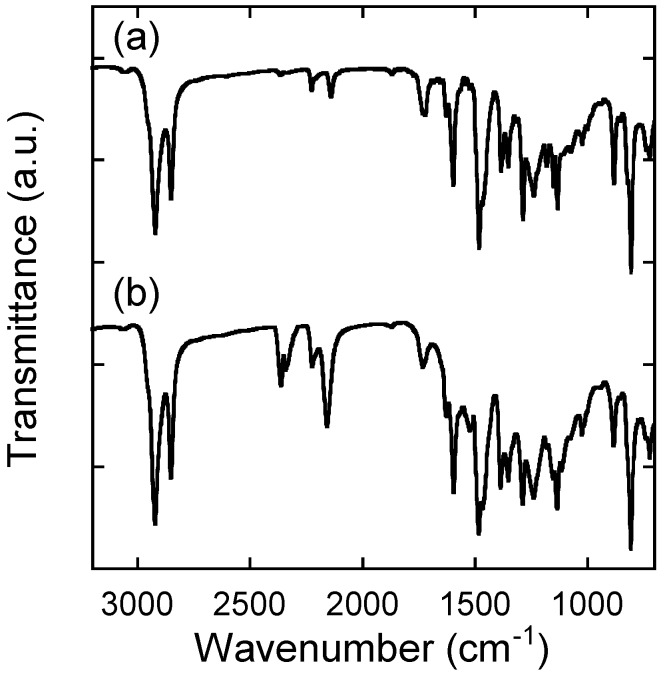
IR spectra (neat) of (**A**) **P1** and (**B**) **P2**.

IR spectra of **P1** and **P2** were similar, except for the alkyne or cyano vibrational peak positions (Figure 3). The precursor polymer **P1** displayed two weak vibrational peaks at 2138.7 and 2224.5 cm^−1^, which are ascribed to the butadiynylene spacers. These peaks existed in the TCNE adducted polymer **P2**, indicating the presence of butadiynylene spacers. **P2** exhibited a new intense peak at 2156.0 cm^−1^, originating from the cyano vibration of the tetracyanobutadiene moieties. This cyano peak position is apparently different from that of TCNE (2257 cm^−1^), suggesting the absence of unreacted TCNE in **P2**.

### 2.3. Thermal Analysis

**Figure 4:**
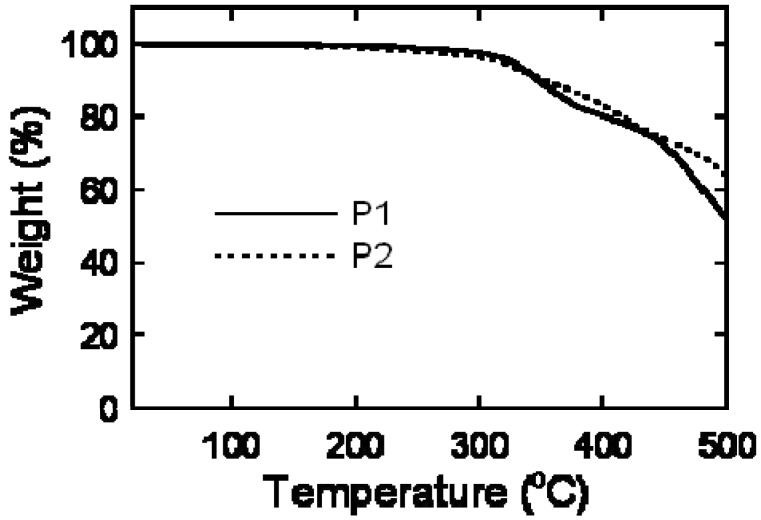
Thermogravimetric analysis of **P1** and **P2** at a heating rate of 10 °C min^−1^ under flowing N_2_.

Thermal stability of the polymers was investigated by thermogravimetric analysis (TGA). It was reported that the postfunctional TCNE addition sometimes enhances the thermal stability of polymers and materials [11,15]. This is also true for this case. The 10% weight loss temperature (*T*_d10%_) of **P1** was 348 °C, while the *T*_d10%_ of **P2** was 353 °C (Figure 4). Furthermore, the soot amount of **P2** at 500 °C was definitely larger than that of **P1**. Although the onset decomposition temperature slightly deteriorated after the TCNE addition, no noticeable decomposition was observed for both polymers at 100 °C. Therefore, this temperature was employed for the postfunctionalization reaction (*vide supra*). It is concluded that the total thermal stability of carbazole-based conjugated polymers was enhanced by the TCNE addition reaction.

### 2.4. Electrochemistry

**Figure 5:**
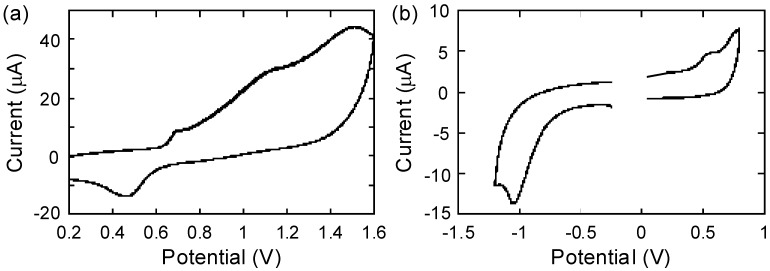
Cyclic voltammograms of (**A**) **P1** and (**B**) **P2** in CH_2_Cl_2_ with 0.1 M (*n*C_4_H_9_)_4_NClO_4_ at the scanning rate of 0.1 V s^−1^ and 20 °C. Potentials *vs.* Fc/Fc^+^.

Cyclic voltammetry measurements were performed in CH_2_Cl_2_ with 0.1 M (*n*C_4_H_9_)_4_NClO_4_ at 20 °C to reveal the redox activities of the polymers and estimate their energy levels. As both polymers showed partial adsorption to the working electrode during the measurements, the potentials recorded in the first cycle were compared. The precursor polymer **P1** displayed the only anodic peak with an onset oxidation potential (*E*_ox,onset_) of 0.64 V (*vs*. Fc/Fc^+^) (Figure 5a). In contrast, the TCNE adducted polymer **P2** exhibited both anodic and cathodic peaks under the same measurement conditions, which are ascribed to the carbazole donor and tetracyanobutadiene acceptor moieties, respectively (Figure 5b). Donor-acceptor type conjugated polymers generally possess narrower band gaps with an elevated HOMO level and a lowered LUMO level, as compared to the single component almost neutral or donor-type polymers. The oxidation of **P2** was thus facilitated compared to **P1**. The *E*_ox,onset_ of **P2** was 0.42 V, which is 0.22 V lower than that of **P1**. Furthermore, a new reduction peak of **P2** appeared with the onset reduction potential *E*_red,onset_ of −0.82 V. The calculated electrochemical band gap of **P2** was 1.24 V, which was almost consistent with the optical band gap (1.46 eV) determined by the *λ*_end_ value of the absorption spectrum in CH_2_Cl_2_ (Table 1). This optical band gap of **P2** was significantly narrower than that of **P1** (2.51 eV). The electronic HOMO and LUMO levels of the polymers were estimated from these data on the basis of the assumption that Fc/Fc^+^ corresponds to −4.80 eV. The precursor polymer **P1** showed the HOMO of −5.44 eV and LUMO of −2.93 eV. After the postfunctional TCNE addition, the HOMO level was slightly elevated to be −5.22 eV. In contrast, the LUMO level dramatically deepened to −3.98 eV. It is anticipated that these energy levels are suitable for p-type semiconducting materials that can be employed in bulk heterojunction organic solar cells with [6,6]-phenyl-C61-butyric acid methyl ester (PCBM) as an n-type semiconductor [29].

**Table 1:** Summary of electrochemical and optical measurements of **P1** and **P2**.

Polymer	*E*_ox,onset_ / V^a^	*E*_red,onset_ / V^a^	Δ|*E*_ox,onset_-*E*_red,onset_| / V	*λ*_end_ / nm^b^	Opt. band gap / eV^c^	HOMO / eV	LUMO / eV
**P1**	0.64	-	-	494	2.51	−5.44^d^	−2.93^e^
**P2**	0.42	−0.82	1.24	950	1.46	−5.22^d^	−3.98^d^

^a^ Measured in CH_2_Cl_2_ with 0.1 M (*n*C_4_H_9_)_4_NClO_4_ at 20 °C. Potentials *vs*. Fc/Fc^+^. ^b^ Measured in CH_2_Cl_2_ at 20 °C. ^c^ Determined by the *λ*_end_ values. ^d^ Determined by the *E*_ox,onset_ or *E*_red,onset_ values based on the assumption of Fc/Fc^+^ = −4.80 eV. ^e^ Calculated from the HOMO and optical band gap.

## 3. Experimental Section

### 3.1. Materials

All reagents were purchased from Kanto, Tokyo Kasei, Wako and Aldrich and used as received. **P1** (*M*_w_ = 15000, *M*_n_ = 7500) and **P3** (*M*_w_ = 12600, *M*_n_ = 9000) were synthesized by the acetylenic oxidation coupling reaction of 3,6-diethynyl-*N*-hexadecylcarbazole and 2,7-diethynyl-*N*-hexadecylcarbazole, respectively, according to a previous report [18].

### 3.2. Measurements

^1^H NMR spectra were measured on a JEOL model AL300 spectrometer at 20 °C. Chemical shifts are reported in ppm downfield from SiMe_4_, using the solvent’s residual signal as an internal reference. The resonance multiplicity is described as s (singlet), br (broad), and m (multiplet). Infrared (IR) spectra were recorded on a JASCO FT/IR-4100 spectrometer. Gel permeation chromatography (GPC) was measured on a JASCO system equipped with polystyrene gel columns using THF as an eluent at a flow rate of 1.0 mL min^−1^. Relative molecular weights were determined by comparison with the calibrated standard polystyrenes. UV-vis spectra were recorded on a JASCO V-670 spectrophotometer, using a 10 mm thick cuvette. Thermogravimetric analysis (TGA) measurements were carried out on a Rigaku TG-DTA ThermoPlus EVO II under flowing nitrogen at the scanning rate of 10 °C min^−1^. Electrochemical measurements of the polymers were carried out at 20 °C in dehydrated CH_2_Cl_2_ containing 0.1 M (*n*C_4_H_9_)_4_NClO_4_ in a classical three-electrode cell. The working, reference, and auxiliary electrodes were a glassy carbon disk electrode (2 mm in diameter), Ag/AgCl/CH_3_CN/(*n*C_4_H_9_)_4_NPF_6_, and a platinum wire, respectively. All potentials are referenced to the ferrocene/ferricinium (Fc/Fc^+^) couple used as an internal standard.

### 3.3. Postfunctionalization with TCNE

In a glass vessel, 15.01 mg (0.03430 mmol repeat unit^−1^) of **P1** was dissolved into 1,2-dichlorobenzene (2 mL). To this solution, 4.39 mg (0.0343 mmol) of TCNE in 1,2-dichloroethane (0.200 mL) was added. Then, the solution was stirred at 100 °C for 48 h. After cooling to 20 °C, the solvent and unreacted TCNE were removed *in vacuo* at 100 °C for 12 h, yielding **P2** (18.15 mg, 71.6% from the gravimetric analysis; 60% from the elemental analysis). ^1^H-NMR (300 MHz, CDCl_3_): δ 0.93 (s, 3n H), 1.34–2.10 (m, 28n H), 3.29 [br s, 2n(1-x) H], 3.68 (br s, 2nx H), 6.86–6.89 (m, 2n H), 7.65 (br s, 2n H), 8.15 ppm (br s, 2n H). IR (KBr): ν 2921.6, 2851.2, 2223.5, 2156.0, 1730.8, 1623.8, 1592.9, 1520.6, 1481.1, 1462.7, 1384.6, 1349.9, 1286.3, 1238.1, 1133.0, 1019.2, 882.3, 804.2, 720.3, 590.1, 512.0 cm^−1^. Elemental analysis calcd for [(C_32_H_39_N)_0.4_(C_38_H_39_N_5_)_0.6_]_n_: C 83.52, H 7.78, N 8.71; found: C 83.18, H 8.27, N 8.55%.

## 4. Conclusions

In summary, it was shown that the postfunctionalization, based on a high yielding addition reaction between electron-rich alkynes and TCNE, is a powerful technique for preparing donor-acceptor alternating polymers. The substitution pattern of carbazole was found to affect the efficiency of the reaction. The 3,6-carbazole-based poly(arylenebutadiynylene) derivative was successfully converted into the donor-acceptor type polymer, while the 2,7-carbazole-based counter polymer did not react with TCNE due to insufficient activation of the alkyne moieties. The resulting donor-acceptor type polymer was characterized by ^1^H NMR, IR, and elemental analysis, and it featured a narrower band gap than the precursor polymer, as revealed by the well defined CT bands in the visible to near-infrared region as well as the potent redox activities. Moreover, the high thermal stability with *T*_d10%_ exceeding 350 °C was achieved by this postfunctionalization. All these features are well suited for applications to p-type semiconducting polymers in bulk heterojunction solar cells.
